# Trade-off between yield and quality driven by artificial domestication in *Gleditsia sinensis*: Phenotypic and functional component variation in a common garden and variety selection

**DOI:** 10.3389/fpls.2026.1914076

**Published:** 2026-07-30

**Authors:** Ganggang Zhang, Ruilin Zhang, Jingyang Feng, Yuxin Jiang, Yingying Chang, Xueying Huo

**Affiliations:** 1 College of Life Sciences, Henan Normal University, Xinxiang, China; 2 Puyang Field Scientific Observation and Research Station for Yellow River Wetland Ecosystem, Puyang, China; 3 Research Center for Ecological Management and Protection of the Yellow River Basin, Xinxiang, China; 4 Henan Academy of Forestry Sciences, Zhengzhou, China

**Keywords:** artificial domestication, common garden, functional components, *Gleditsia sinensis*, phenotypic traits, variety selection

## Abstract

*Gleditsia sinensis* is an ecological and economic native tree species, yet previous origin-based studies suffer from genotype-by-environment (G×E) interactions, lacking systematic comparisons of morphology-metabolism associations between cultivated and wild types without environmental bias. To address this, pod/seed morphology and key quality traits of 15 *G. sinensis* germplasms (one wild control, 14 cultivars) were evaluated in a common garden, integrating analysis of variance (ANOVA), random forest, correlation, principal component analysis (PCA), and cluster analyses. Cultivars significantly outperformed the wild type in pod dry weight, 1000-seed weight, and total saponins, whereas the wild type accumulated significantly higher total polyphenols and flavonoids. Phenotypic variation was greater in pods than in seeds, and morphological variation of both pods and seeds exceeded that of nutritional quality, indicating that the latter exhibits higher genetic stability. Random forest identified seeds per pod, 1000-seed weight, seed dry weight, and pod dry weight as the top predictors for nutritional quality. Notably, larger and heavier structures correlated negatively with total polyphenols, flavonoids, total sugar, and crude fiber, but positively with pectin, ash, soluble protein, and total soluble sugar, suggesting a potential growth-defense trade-off. Multidimensional evaluation categorized the germplasms into four distinct clusters (Cluster I: YJ5, YJ4, YJ6; Cluster II: JD2, JD3, JD9, JD10, YJ3; Cluster III: CK, JD1; Cluster IV: JD7, YJ10, YJ2, JD11, YL1). Within these, JD9, YJ2, and YJ10 demonstrated balanced agronomic and nutritional performance, making them promising comprehensive cultivars. Meanwhile, the wild type (CK) and JD1 are highlighted as crucial donor parents for nutritional improvement, whereas YJ5 and YJ6 are ideal materials for enhancing morphological traits. Ultimately, this study elucidates the potential growth-defense trade-off during woody plant domestication, providing a scientific basis for the differentiated directional breeding and the utilization of wild genes in *G. sinensis*.

## 1 Introduction

*Gleditsia* Linn is widely distributed across the globe, and *Gleditsia sinensis* Lam. is a perennial deciduous tree within the Leguminosae family (Xiao et al., 2025; Liu et al., 2024). As a multifunctional ecological and economic native tree species unique to China (Lv et al., 2025; Liu et al., 2024), *G. sinensis* can potentially be cultivated across over 50.0% of the national land area. It possesses desirable biological traits, including well-developed root systems, nitrogen fixation capacity, strong resistance to pollution, and high stress tolerance (Zhang et al., 2025a). Serving as a pioneer tree for vegetation restoration on rocky mountains and difficult sites (Liu et al., 2024), it is also a preferred species for the National Reserve Forest Project and one of the Ten Recommended Tree Species for ecological management in the Yellow River Basin, playing an irreplaceable role in regional ecological barriers (Zhang et al., 2025a). *G. sinensis* is a versatile woody plant featured with homology of medicine and food (Gao et al., 2016; Luo et al., 2025; Zhang et al., 2017). The fruits can serve as medicine, food, cosmetics, and natural raw materials (Lian and Zhang, 2013); the seeds contain important vegetable gum (Zhu et al., 2014); and the thorns contain flavonoid glycosides and phenols (Gao et al., 2016). Modern pharmacological studies demonstrate that its pods and seeds are rich in triterpenoid saponins, polyphenols, flavonoids, and dietary fiber (Zhang et al., 2016; Lian and Zhang, 2013; Qin et al., 2023; Li et al., 2016). These secondary metabolites exert remarkable effects on eliminating phlegm, antibacterial activities, and blood lipid regulation (Qin et al., 2023; Li et al., 2016). With the vigorous development of the traditional Chinese medicine (TCM) holistic health industry (Zhang et al., 2017), market demand for high-activity *G. sinensis* raw materials has surged. Recently, the *G. sinensis* industry has grown significantly in northern China, establishing it as a major new economic forest plant with a rapidly expanding planting area. However, in actual cultivation, pod and seed yields have generally been unstable and relatively low, posing a serious challenge for the industry’s long-term development and limiting its widespread promotion (Lv et al., 2025; Zhang et al., 2025a). To address this, Henan Province, a core production area with abundant natural resources (Zhang et al., 2025a; Lan and Gu, 2006), has bred new fruit-oriented cultivars like Jindou and Yujia (Zhou et al., 2022), transforming the industry from wild collection to intensive cultivation. Despite these breeding advancements, the lack of systematic research on phenotypic diversity hinders the precise selection of superior varieties, restricting the industry’s high-quality development (Luo et al., 2025).

However, precise germplasm evaluation and directional breeding lag behind. Expression of a phenotype is a function of the genotype, the environment, and their interaction (G × E) (De Leon et al., 2016). Plant primary and secondary metabolites are not only a useful array of natural products but also an important part of the plant defense system (Yang et al., 2018) and the material basis of clinically curative effects (Li et al., 2020a). Their synthesis and accumulation are complex, affected by internal genetic circuits and external environmental factors (Li et al., 2020a). Previous studies on *G. sinensis* traits and active components were mostly based on cross-regional comparisons (Luo et al., 2025; Xiao et al., 2024), inevitably incorporating environmental noise and leading to false positives in correlation analyses (Stotz et al., 2025; Matesanz et al., 2019; Valladares et al., 2007). For instance, environmental stresses can lead to significant changes in secondary metabolites and chemical compositions of the essential oils from some medicinal plants, affecting medicinal and aromatic properties (Laftouhi et al., 2023). Similarly, saponin content is variable and influenced by the surrounding environment, substantially impacting the quality of medicinal plants (Szakiel et al., 2010). Thus, a high saponin content might reflect local stresses rather than genetic superiority. This environmental confounding masks true genotypic effects and makes conclusions difficult to integrate. Furthermore, current breeding is biased toward yield traits like larger pods and heavier seeds (Zeng et al., 2025; Wang et al., 2020; Luo et al., 2025), neglecting the synergistic improvement of medicinal quality. Without environment-independent studies on plant physical-metabolites associations, breeders lack indirect selection indices for internal quality, leaving high-quality selection in a blind state.

A common garden is a uniform setting where traits can be characterized without the confounding influences of environmental variation (Huxman et al., 2021). Common garden experiments are precious for studying adaptive potential without the confounding effect of phenotypic plasticity (De Villemereuil et al., 2020). These experiments test how heritable traits are shaped by natural selection, governed by tradeoffs where increasing the fitness contribution of one trait compromises another (De Villemereuil et al., 2020; Schwinning et al., 2022). To survive in hostile habitats, plants manage resources to balance development and defense, setting up a trade-off (Figueroa-Macías et al., 2021). Human selection on wild populations mostly favored specific traits during domestication, but this direct selection also altered independent traits not directly desired (Singh and Van Der Knaap, 2022). Domestication reshuffles carbon allocation: strong directional selection on yield transfers resources from defense (the secondary metabolites like polyphenols) to growth, triggering the growth-defense trade-off (Figueroa-Macías et al., 2021; Singh and Van Der Knaap, 2022). For *G. sinensis*, whose core economic value lies in metabolites, has domestication also reshaped its resource allocation? Do cultivated varieties increase pod size at the cost of diluting or suppressing functional components? These core domestication questions remain empirically untested in *G. sinensis*.

Based on the above, this study utilized a *G. sinensis* common garden in Henan to systematically compare phenotypic traits and nutritional quality of 14 main cultivars (covering Jindou, Yujia, and Yulin series) using one wild population as a control. We propose the following core hypotheses: (1) Artificial domestication has not only altered the external morphology of *G. sinensis*, but also potentially influence its resource allocation strategy, manifesting as a potential negative correlation between reproductive growth (yield) and secondary metabolism (functional components); (2) Under common garden conditions, there are stable and reliable associations between external physical indicators and internal chemical qualities, which can serve as predictive indicators for early breeding. This study aims to reveal the variation patterns and association mechanisms of *G. sinensis* traits, clarify the differentiation characteristics between wild and cultivated germplasm, and provide a solid theoretical basis for the screening of high-quality germplasm, directional breeding (yield-oriented vs. functional-oriented), and the mining and utilization of excellent wild genes.

## 2 Materials and methods

### 2.1 Overview of the study area

**Figure 1:**
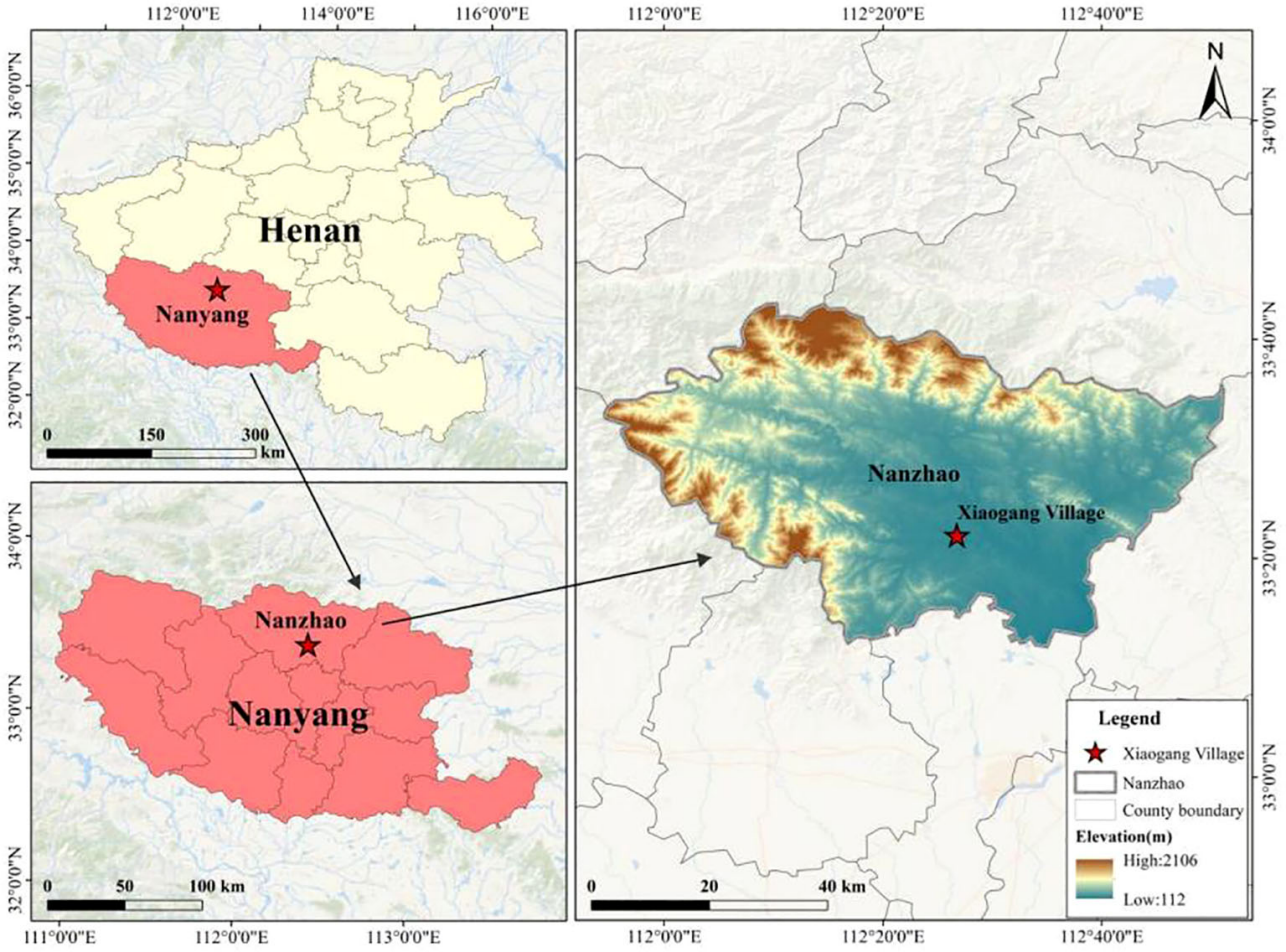
Overview of the study area. The study site is located in Xiaogang Village, Nanzhao County, northern Nanyang City, southwestern Henan Province.

Nanzhao County (33°12′–33°43′ N, 111°55′–112°51′ E) is located in southwestern Henan Province (**Figure 1**). The region features a northern subtropical monsoon continental climate situated at a transitional edge, with a mean annual temperature of 14.8 °C, and a mean annual precipitation of 869.3 mm. The soils are primarily yellow-brown soil, brown soil, and fluvo-aquic soil, with a pH of 5.5–7.5. This region boasts abundant plant resources and serves as an important ecological barrier. Furthermore, Nanzhao County is the largest distribution center for *G. sinensis* seeds in China. In recent years, through industry-research cooperation with the Henan Academy of Forestry Sciences, several new fruit-type *G. sinensis* cultivars (e.g., ‘Jindou 1’, ‘Jindou 2’, ‘Yujia 2’, ‘Yujia 3’) have been bred. Therefore, this region is highly representative of the breeding and cultivation of improved *G. sinensis* varieties.

### 2.2 Experimental plant material

**Figure 2:**
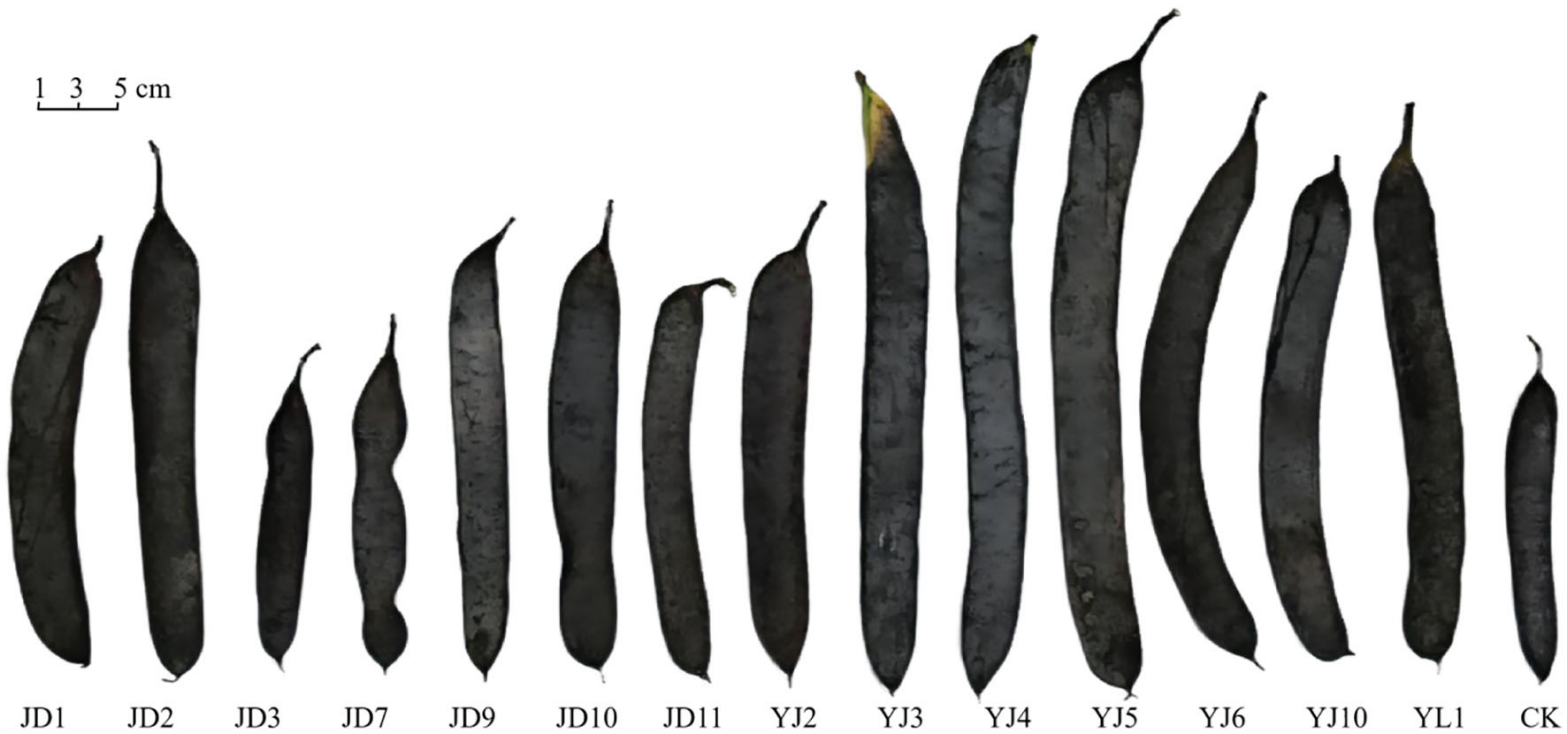
Pods of different *G. sinensis* cultivars. Seven Jindou series cultivars (JD1, JD2, JD3, JD7, JD9, JD10, JD11), six Yujia series cultivars (YJ2, YJ3, YJ4, YJ5, YJ6, YJ10), one Yulin series cultivar (YL1), and wild *G. sinensis* as the control (CK).

In October 2025, plant materials were collected from the Nanzhao *G. sinensis* provenance common garden (33°22′2.02″N, 112°27′7.37″E) of the Henan Academy of Forestry Sciences, which is located in Xiaogang Village, Nanzhao County. The garden has an altitude of 181 m, with yellow-brown soil and a pH of 6.4. This site is characterized by consistent soil physicochemical properties and unified field water and fertilizer management. A wild *G. sinensis* population (CK) was collected as the control, and a total of 14 cultivated varieties were selected as the research subjects, comprising 7 Jindou series varieties (JD1, JD2, JD3, JD7, JD9, JD10, and JD11), 6 Yujia series varieties (YJ2, YJ3, YJ4, YJ5, YJ6, and YJ10), and 1 Yulin series variety (YL1) (**Figure 2**). Sampling followed a two-step strategy combining purposive and random sampling strategies. First, a purposive sampling strategy was employed to select representative sample plants showing no obvious pests, diseases, or mechanical damage, and spaced more than 20 m apart (to minimize kinship correlation among samples). For each variety, four individual plants were selected as the experimental units, serving as four biological replicates (*n* = 4). From each plant, three intact pods of consistent maturity were collected from each of the four cardinal directions (east, south, west, and north). This purposive selection ensured that only healthy, mature, and undamaged pods were included in the valid sample pool, yielding a total of 48 pods per variety (4 plants × 4 directions × 3 pods). These pods were treated as sub-samples to capture intra-individual variability. After numbering and weighing, the samples were snap-frozen in liquid nitrogen and transported to the laboratory for subsequent trait measurement and analysis. Second, a random sampling strategy was applied to allocate these purposively selected samples: 35 pods were randomly selected for morphological trait evaluations, and 3 pods were randomly selected, specifically ensuring that each was collected from a different individual tree, to provide 3 biological replicates (*n* = 3) for nutritional quality indicator determination. The remaining 10 pods were retained as backup samples in case of unexpected damage or assay failure.

### 2.3 Morphological trait measurement

The 35 pods previously selected from each variety were used to measure the morphological traits of the pods and seeds. Pod length (PL), pod width (PW), and pod thickness (PT) were measured using a ruler with a precision of 0.01 cm. Seed length (SL) and seed width (SW) were measured using a vernier caliper with a precision of 0.02 mm. Additionally, pod length-width product (PLWP), pod length-width ratio (PLWR), seed length-width product (SLWP), and seed length-width ratio (SLWR) were calculated. Simultaneously, the number of seeds within each pod was recorded as seeds per pod (SPP). Fresh pod weight (FPW) and dry pod weight (DPW) were measured using an electronic balance with a precision of 0.001 g, and pod moisture content (PMC) and seed moisture content (SMC) were subsequently calculated. Furthermore, 100 seeds were randomly selected to measure their fresh and dry weights; this sampling was repeated 10 times, and the average value was converted to the thousand-seed weight (TSW). All morphological measurements were first averaged at the individual tree level to generate a single mean value per plant, ensuring that the four individual plants served as the true biological replicates (*n* = 4) for subsequent statistical analyses.

### 2.4 Nutritional quality measurement

To evaluate the quality of *G. sinensis* germplasm, three samples were randomly selected from the remaining 13 samples stored at -80°C. As defined in Section 2.2, these three samples were derived from three different individual trees per variety, serving as three independent biological replicates (n = 3). These were ground into a fine powder in liquid nitrogen to maximize their preservation of original physicochemical properties, thereby ensuring the reliability and stability of the analytical data. The nutritional quality indicators of pods and seeds were determined following standard analytical protocols (**Table 1**).

**Table 1:** Determination methods for nutritional quality indicators of pod and seed.

Indicator	Abbreviation	Method	References
Soluble Protein	SP	Coomassie Brilliant Blue colorimetric method	(Wang and Huang, 2015)
Crude Fat	CFat	Soxhlet extraction method	(State Administration for Market Regulation, 2026)
Total Starch	TSt	Acid hydrolysis method	(National Food Safety Standard: Determination of Starch in Food, 2023)
Total Sugar	TSu	Phenol-sulfuric acid colorimetric method	(Standardization Administration of China, 2009)
Total Soluble Sugar	TSS	Anthrone-sulfuric acid colorimetric method	(Wang and Huang, 2015)
Total Kjeldahl Nitrogen	TKN	Micro-Kjeldahl method	(Wang and Huang, 2015)
Crude Fiber	CF	Acid-base washing method	(Wang and Zheng, 1990)
Ash	Ash	High-temperature dry ashing method	(Standardization Administration of the People's Republic of China, 2018)
Pectin	Pec	Carbazole colorimetric method	(Ministry of Agriculture of the People's Republic of China, 2011)
Total Polyphenols	TP	Folin-phenol method	(Li et al., 2008)
Total Saponins	TSs	Spectrophotometric method	(Guangdong Association for Food and Drug Evaluation & Certification Technology, 2022)
Flavonoids	Flav	Spectrophotometric method	(Wei et al., 2003)
Ascorbic Acid	AA	2,6-Dichloroindophenol titration method	(National Health and Family Planning Commission, 2016)
Catalase activity	CAT	Ultraviolet spectrophotometric method	(Wang and Huang, 2015)
Peroxidase activity	POD	Guaiacol colorimetric method	(Wang and Huang, 2015)

### 2.5 Statistical analysis

All statistical analyses were performed in the R language (Version 4.5.3). Initially, the mean, standard deviation, and coefficient of variation were calculated for each morphological trait and nutritional quality indicator across all cultivars. Additionally, to evaluate the overall variation of a specific cultivar within a given trait category (e.g., pod, seed, or nutritional traits), the average CV was calculated as the mean of the CVs across all individual traits within that category. One-way ANOVA and Tukey’s HSD *post-hoc* test (α = 0.05) were conducted using the stats and agricolae packages to test the significance of differences among cultivars. Spearman rank correlation analysis was applied to assess the correlations among all indicators. Additionally, a Random Forest (RF) regression analysis was constructed using the randomForest package to evaluate the importance of the 15 pod and seed morphological traits (predictor variables) in predicting the 15 nutritional quality indicators (response variables). The model was parameterized with ntree = 1000 and mtry = 5 (the default value of p/3 for regression). The percent increase in mean squared error (%IncMSE) was used as the variable importance metric. Instead of a traditional K-fold cross-validation, the inherent Out-Of-Bag (OOB) sampling mechanism of the RF algorithm was utilized to validate model robustness, from which model performance statistics, including the OOB error, explained variance (*R*^2^), and root mean square error (RMSE), were derived.

All morphological and nutritional quality indicators were first summarized as cultivar mean values across biological replicates, and the resulting cultivar-mean matrix was used as the analytical unit. Each indicator matrix was z-score standardized (centered at mean zero and scaled to unit variance), and principal component analysis (PCA) was performed on the standardized data using the prcomp function, which is mathematically equivalent to PCA on the correlation matrix of the original variables. Independent principal components were extracted based on the standard criteria that principal components with eigenvalues greater than 1 and a cumulative variance contribution rate exceeding 80% can sufficiently capture most information contained in the original variables.

Therefore, composite morphological trait scores (M) and nutritional quality scores (N) were constructed using the variance contribution rate of the extracted principal components as weights. The formula for the composite score is:


$$M\left(or\;N\right)=\sum_{i=1}^{n}w_{i}\times s_{i}$$

Where *w*_1_, *w*_2_, …, *w_k_* are the weights represented by the variance contribution rates of the extracted principal components, and *s*_1_, *s*_2_, …, *s_k_* are the principal component scores calculated from the loading (score coefficient) matrix. After calculating the M and N values for each cultivar using the membership function method (Luo et al., 2025), a comprehensive evaluation value (C) was calculated for each cultivar based on the mean of the morphological (M) and nutritional quality (N) scores.

Finally, hierarchical cluster analysis (based on Euclidean distance and the Ward.D2 method) was performed using the stats package on the membership degrees M and N in a two-dimensional space to classify different cultivar groups.

## 3 Results and analysis

### 3.1 Variation in pod and seed morphological traits of different cultivars

The dry pod weight (DPW), pod length (PL), pod width (PW), pod length-width product (PLWP), dry seed weight (DSW), thousand-seed weight (TSW), seed width (SW), and seed length-width product (SLWP) of the Yujia and Yulin series cultivars were significantly higher than those of the wild control (CK) (*P* < 0.05), whereas their seed length-width ratio (SLWR) was significantly lower than that of CK (*P<* 0.05) (**Table 2**). The TSW of the Jindou, Yujia, and Yulin series cultivars was also significantly higher than that of CK (*P* < 0.05). Within the Jindou series, JD1 exhibited significantly higher DPW and PT than the other cultivars within the same series, while JD2 showed significantly higher SL and SLWP. Furthermore, the DPW and seed moisture content (SMC) of JD3 were significantly higher than those of the Yujia and Yulin series cultivars. Within the Yujia series, YJ6 displayed significantly higher pod thickness (PT), TSW, seed length (SL), and SLWP compared to its counterparts. Notably, the PL, PW, PLWP, DSW, TSW, and SL of both YJ5 and YJ6 were significantly higher than those of all Yulin series cultivars. In the Yulin series, YL1 achieved the maximum PT (1.59 ± 0.14 cm), which was significantly higher than that of all Yujia series cultivars and CK.

**Table 2:** Pod and seed morphology of different cultivars (mean ± SD).

Sp	DPW/g	PMC/%	SPP	PL/cm	PW/cm	PLWP/cm^2^	PLWR	PT/cm	DSW/g	SMC/%	TSW/g	SL/cm	SW/cm	SLWP/cm^2^	SLWR
JD1	15.37 ± 4.09 gh	14.56 ± 3.39 abc	10.44 ± 4.65 e	16.56 ± 2.16 e	2.57 ± 0.25 fg	42.84 ± 8.69 h	6.45 ± 0.74 f	1.27 ± 0.11 cd	4.7 ± 1.92 hi	9.04 ± 4.21 cdef	461 ± 59.27 fg	1.1 ± 0.07 f	0.88 ± 0.08 e	0.97 ± 0.13 f	1.26 ± 0.09 ef
JD2	27.44 ± 4.2 cd	14.77 ± 3.14 abc	17.74 ± 3.83 ab	23.43 ± 2.02 bcd	2.95 ± 0.17 c	69.1 ± 7.18 ef	7.97 ± 0.81 cde	1.36 ± 0.08 bc	12.4 ± 2.73 bcd	13.29 ± 2.17 ab	699.86 ± 52.92 b	1.42 ± 0.06 b	1.01 ± 0.05 c	1.43 ± 0.11 b	1.41 ± 0.08 cde
JD3	42.41 ± 10.2 a	15.07 ± 2.50 ab	17.26 ± 3.7 ab	23.98 ± 3 bcd	3.32 ± 0.21 ab	80.06 ± 13.55 cd	7.21 ± 0.72 ef	1.58 ± 0.09 a	12.52 ± 2.76 bc	13.99 ± 5.18 a	722.4 ± 64.71 b	1.3 ± 0.09 cd	1.01 ± 0.13 bc	1.32 ± 0.19 c	1.22 ± 0.28 f
JD7	21.23 ± 8.51 efg	11.27 ± 2.84 de	15.57 ± 8.35 bcd	23.15 ± 5.81 bcd	2.87 ± 0.22 cd	67.1 ± 19.35 ef	8.03 ± 1.84 cde	1.05 ± 0.12 f	5.96 ± 3.16 gh	9.52 ± 4.90 cdef	398.01 ± 91.47 g	1.1 ± 0.1 f	0.69 ± 0.08 f	0.76 ± 0.12 g	1.63 ± 0.29 ab
JD9	24.59 ± 3.25 cdef	13.33 ± 3.18 bcd	19.86 ± 3.12 a	25.65 ± 2.19 b	2.89 ± 0.23 cd	74.27 ± 9.46 cdef	8.91 ± 0.91 ab	1.25 ± 0.13 de	10.36 ± 1.78 cde	9.7 ± 4.03 cde	521.31 ± 85.09 ef	1.32 ± 0.11 cd	0.91 ± 0.06 de	1.2 ± 0.15 de	1.46 ± 0.09 cd
JD10	29.66 ± 5.66 c	13.81 ± 4.13 abc	17.09 ± 4.46 abc	25.55 ± 2.57 b	3.24 ± 0.16 ab	82.98 ± 10.57 c	7.89 ± 0.75 de	1.21 ± 0.1 de	8.24 ± 2.51 efg	8.91 ± 5.11 cdef	476.22 ± 46.1 ef	1.15 ± 0.09 ef	0.88 ± 0.07 e	1.02 ± 0.14 f	1.31 ± 0.08 def
JD11	20.01 ± 3.48 fg	15.78 ± 3.66 a	16.94 ± 2.92 abc	20.62 ± 2.14 d	2.72 ± 0.16 def	56.05 ± 6.85 g	7.61 ± 0.88 e	1.36 ± 0.09 bc	9.16 ± 2.1 ef	12.23 ± 3.43 abc	537.61 ± 70.78 de	1.29 ± 0.06 d	0.97 ± 0.08 cd	1.25 ± 0.13 cde	1.33 ± 0.11 def
YJ2	26.2 ± 4.04 cde	14.85 ± 2.79 abc	17 ± 4.78 abc	22.43 ± 1.86 cd	3.40 ± 0.21 a	76.45 ± 9.52 cde	6.61 ± 0.52 f	1.23 ± 0.25 de	10.17 ± 2.93 de	11.26 ± 3.07 bcd	603.16 ± 69.98 c	1.32 ± 0.07 cd	0.98 ± 0.07 cd	1.3 ± 0.12 cd	1.35 ± 0.12 def
YJ3	27.19 ± 6.68 cd	9.41 ± 2.88 e	17.09 ± 5.39 abc	24.96 ± 3.92 bc	2.84 ± 0.22 cde	71.33 ± 14.87 def	8.78 ± 1.15 bc	1.3 ± 0.16 cd	10 ± 3.46 e	7.49 ± 3.82 ef	585.12 ± 103.78 cd	1.21 ± 0.08 e	0.98 ± 0.08 c	1.19 ± 0.16 e	1.23 ± 0.09 f
YJ4	28.8 ± 7.84 cd	13.78 ± 2.64 abc	13.31 ± 6.1 cde	29.6 ± 4.01 a	3.18 ± 0.15 b	94.28 ± 13.75 ab	9.31 ± 1.31 ab	1.08 ± 0.14 f	9.31 ± 4.39 ef	8.65 ± 2.76 def	700.93 ± 71.01 b	1.37 ± 0.06 bc	1.08 ± 0.07 a	1.48 ± 0.09 b	1.25 ± 0.15 f
YJ5	35.05 ± 8.19 b	8.45 ± 1.59 e	20.37 ± 4.81 a	30.03 ± 4.4 a	3.21 ± 0.19 ab	96.67 ± 16.66 a	9.36 ± 1.35 ab	1.28 ± 0.11 cd	14.08 ± 3.91 ab	6.22 ± 2.76 f	689.74 ± 137.07 b	1.3 ± 0.07 cd	0.99 ± 0.07 c	1.29 ± 0.12 cde	1.31 ± 0.18 def
YJ6	39.01 ± 6.59 ab	13.01 ± 2.31 bcd	17.89 ± 4 ab	28.38 ± 3.45 a	2.95 ± 0.2 c	83.9 ± 13.93 bc	9.64 ± 1.04 a	1.45 ± 0.07 b	14.97 ± 3.67 a	10.49 ± 3.88 bcd	834.9 ± 61.07 a	1.62 ± 0.08 a	1.08 ± 0.05 ab	1.74 ± 0.13 a	1.5 ± 0.09 bc
YJ10	22.05 ± 6.26 defg	11.81 ± 2.42 cde	15.36 ± 6.28 bcde	24.31 ± 2.82 bcd	3.07 ± 0.16 bc	74.87 ± 10.83 cdef	7.91 ± 0.82 cde	1.1 ± 0.14 ef	6.94 ± 3.2 fgh	8.92 ± 3.58 def	442.42 ± 37.04 fg	1.18 ± 0.03 ef	0.87 ± 0.04 e	1.02 ± 0.05 f	1.35 ± 0.06 cdef
YL1	28.54 ± 4.63 cd	11.31 ± 2.38 de	14.82 ± 3.18 bcde	22.64 ± 1.85 cd	2.66 ± 0.18 ef	60.28 ± 6.43 fg	8.54 ± 0.89 bcd	1.59 ± 0.14 a	7.21 ± 1.76 fg	9.00 ± 3.55 def	487.15 ± 59.26 ef	1.17 ± 0.05 ef	1.03 ± 0.04 abc	1.21 ± 0.08 de	1.14 ± 0.05 f
CK	11.77 ± 4.6 h	13.63 ± 3.83 abc	11.8 ± 3.57 de	17.51 ± 4.25 e	2.41 ± 0.51 g	44.07 ± 21.39 h	7.26 ± 0.69 ef	1.04 ± 0.15 f	3.26 ± 1.07 i	9.81 ± 4.23 cde	283.92 ± 61.83 h	1.16 ± 0.12 ef	0.67 ± 0.1 f	0.78 ± 0.12 g	1.73 ± 0.45 a

Sp represents cultivars; JD1, JD2, JD3, JD7, JD9, JD10, JD11 are Jindou series cultivars, YJ2, YJ3, YJ4, YJ5, YJ6, YJ10 are Yujia series cultivars, YL1 is Yulin series cultivar, and CK is the wild species as the control; DPW, dry pod weight; PMC, pod moisture content; SPP, seeds per pod; PL, pod length; PW, pod width; PLWP, pod length-width product; PLWR, pod length-width ratio; PT, pod thickness; DSW, dry seed weight; SMC, seed moisture content; TSW, thousand-seed weight; SL, seed length; SW, seed width; SLWP, seed length-width product; SLWR, seed length-width ratio. Values in the table are expressed as the mean ± standard deviation. Different lowercase letters in the same column indicate significant differences (P< 0.05). The same below.

**Figure 3:**
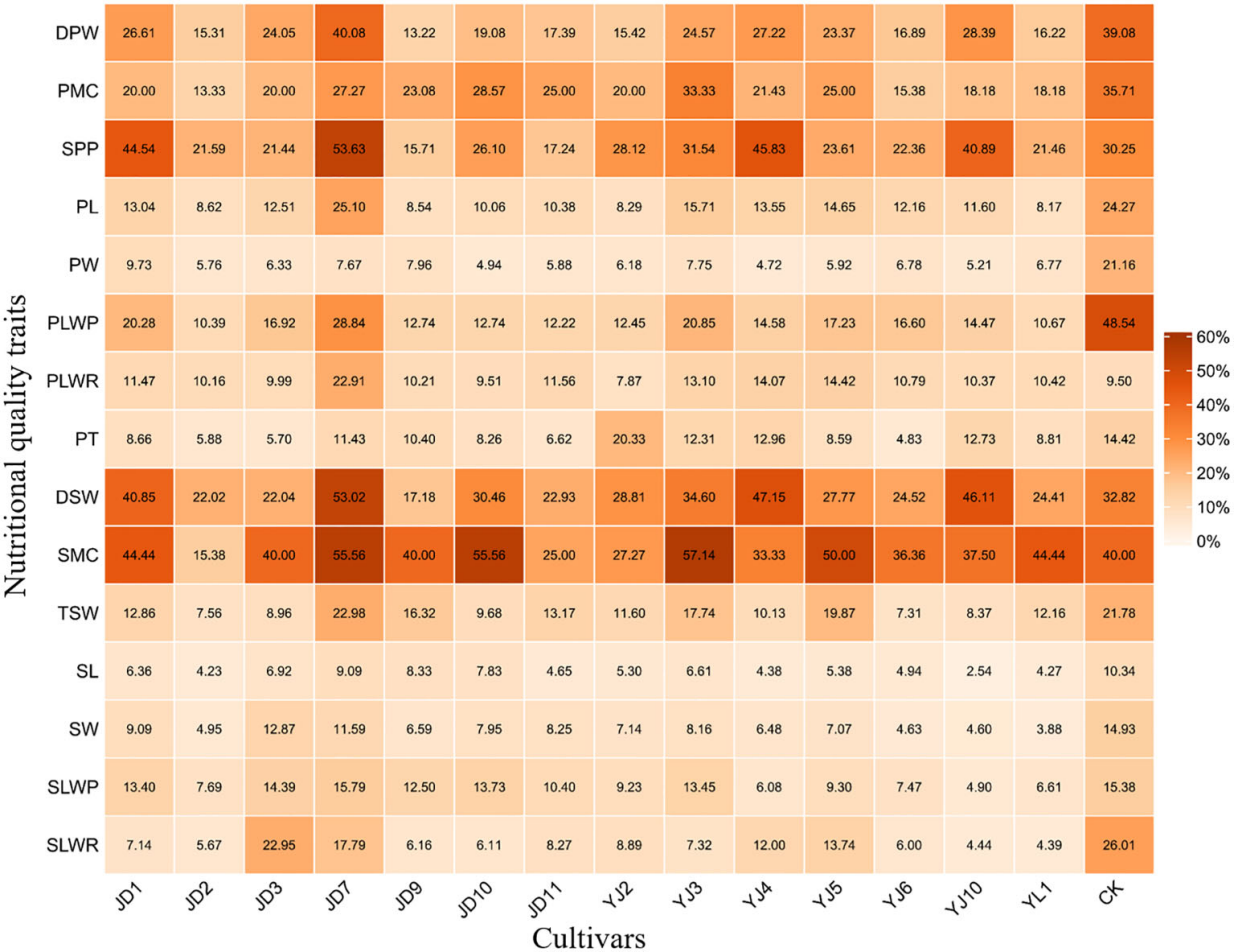
Coefficient of variation of pod and seed morphological traits.

For pod morphology across the cultivars (**Figure 3**), the coefficient of variation (CV) was generally high for seeds per pod (SPP) (29.62%), DPW (23.13%), and pod moisture content (PMC) (22.96%), but relatively low for PW (7.52%) and PT (10.13%). The wild control (CK) exhibited the highest average CV across pod traits (27.87%), with the CVs of DPW, PMC, PL, PW, and PLWP all exceeding 20%. Within the Jindou series, JD7 showed the maximum average CV (27.12%), with the CVs of DPW, PMC, SPP, PL, PLWP, and pod length-width ratio (PLWR) all exceeding 20.00%, whereas JD2 exhibited the minimum average CV at only 11.38%. In the Yujia series, YJ3 and YJ4 displayed relatively high average CVs of 19.89% and 19.30%, respectively. In the Yulin series, YL1 exhibited the lowest average CV at only 12.59%.

Regarding seed morphological variation across the cultivars, DSW (31.65%) and SMC (40.13%) exhibited relatively high CVs, whereas SL (6.08%) and SW (7.88%) showed relatively low CVs. The wild control (CK) displayed substantial average CV in seed morphology (23.04%), with the CVs of SL, SW, SLWP, and SLWR being the highest among all accessions. Within the Jindou series, JD7 exhibited the highest average CV (26.55%), prominently driven by DSW (53.02%), SMC (55.56%), and TSW (22.98%). In the Yujia series, YJ3 showed the highest average CV (20.72%).

Overall, the average CV in seed size and morphological traits (8.78%) was lower than that of pod traits (12.59%), indicating greater variability in pod characteristics and a higher stability in seed traits. Among all germplasms, JD7 exhibited the highest overall average CV in pod and seed morphology (26.85%), followed by CK (25.61%) and YJ3 (20.28%), whereas JD2 showed the lowest overall average CV (10.57%). Additionally, the average CVs in pod and seed morphology were relatively similar among YJ6 (13.13%), JD11 (13.26%), YL1 (13.39%), and JD9 (13.93%).

### 3.2 Variation in pod and seed nutritional quality traits of different cultivars

The total polyphenols (TP) and flavonoids (Flav) contents of CK were significantly higher than those of all cultivars in the Jindou, Yujia, and Yulin series (P< 0.05) (**Table 3**). Within the Jindou series, JD3 exhibited significantly higher crude fat (CFat) and pectin (Pec) contents than the other cultivars within the same series, but its TP and catalase (CAT) contents were significantly lower. Meanwhile, JD2 showed a significantly higher TSs content than its counterparts, whereas its CFat content was significantly lower. In the Yujia series, YJ2 displayed significantly higher TSS, TP, and CAT contents compared to the other cultivars within the same series; YJ4 had a significantly higher crude fiber (CF) content, but its soluble protein (SP), total soluble sugar (TSS), TP, total saponins (TSs), and CAT contents were significantly lower; YJ6 exhibited significantly higher SP, Ash, and peroxidase (POD) contents, whereas its total sugar (TSu) content was significantly lower. In the Yulin series, YL1 achieved the maximum Ash content (0.20 ± 0.01 mg/g), which was significantly higher than that of CK.

**Table 3:** Pod and seed nutritional quality characteristics of different cultivars (Mean ± SD).

Sp	SP/(mg/g)	CFat/%	TSt/(mg/g)	TSu/(mg/g)	TSS/(mg/g)	TKN/(mg/g)	CF/(mg/g)	Ash/(mg/g)	Pec/(mg/g)	TP/(mg/g)	TSs/(mg/g)	Flav/(mg/g)	AA(mg/g)	CAT/U	POD/U
JD1	22.97 ± 0.43 h	7.05 ± 0.27 cd	342.39 ± 8.31 a	294.12 ± 6.92 ab	156.96 ± 0.62 ef	28.47 ± 2.12 d	30.69 ± 0.99 f	0.11 ± 0.00 efgh	22.13 ± 1.05 e	8.09 ± 0.20 ef	59.63 ± 1.78 d	20.57 ± 0.72 c	0.28 ± 0.00 ab	148.30 ± 0.43 b	0.67 ± 0.05 h
JD2	29.58 ± 0.56 cd	5.00 ± 0.19 i	307.17 ± 2.04 cd	214.73 ± 2.12 hi	174.44 ± 0.23 bc	38.84 ± 1.59 a	26.86 ± 0.40 g	0.17 ± 0.01 abc	36.38 ± 1.21 c	8.55 ± 0.21 de	90.72 ± 2.29 a	12.31 ± 0.20 fg	0.29 ± 0.01 ab	128.76 ± 17.09 c	1.34 ± 0.03 g
JD3	26.96 ± 0.75 efg	8.37 ± 0.33 a	287.14 ± 10.10 de	253.27 ± 3.63 ef	180.04 ± 1.16 ab	35.66 ± 3.02 abc	24.88 ± 0.89 g	0.14 ± 0.01 cde	57.99 ± 2.85 a	4.85 ± 0.10 h	55.85 ± 0.75 d	11.06 ± 0.39 g	0.19 ± 0.00 c	55.51 ± 0.28 g	3.36 ± 0.03 d
JD7	25.25 ± 0.49 g	6.31 ± 0.10 efg	244.50 ± 5.14 gh	226.69 ± 5.50 gh	150.19 ± 2.96 fg	28.43 ± 1.77 d	33.99 ± 1.35 cde	0.09 ± 0.00 h	25.74 ± 0.98 de	9.59 ± 0.28 c	57.38 ± 0.92 d	24.26 ± 1.02 b	0.19 ± 0.00 c	148.11 ± 1.30 b	1.66 ± 0.04 f
JD9	28.43 ± 0.83 cde	6.8 ± 0.19 cde	238.79 ± 6.51 h	213.89 ± 5.61 hi	84.95 ± 2.39 j	37.98 ± 2.00 a	34.82 ± 0.70 bcd	0.13 ± 0.00 ef	25.43 ± 1.24 de	8.72 ± 0.24 d	35.74 ± 1.24 f	19.61 ± 0.40 cd	0.09 ± 0.00 d	94.49 ± 0.43 de	3.48 ± 0.05 cd
JD10	30.28 ± 0.37 bc	6.08 ± 0.18 fgh	318.50 ± 3.88 bc	266.26 ± 9.16 de	165.06 ± 3.42 de	38.87 ± 0.62 a	31.26 ± 1.01 ef	0.16 ± 0.01 bcd	24.44 ± 1.13 de	9.73 ± 0.11 c	70.66 ± 1.85 b	24.57 ± 0.61 b	0.29 ± 0.01 ab	103.46 ± 0.33 de	1.74 ± 0.01 f
JD11	27.28 ± 0.47 ef	7.77 ± 0.28 ab	298.96 ± 2.40 cde	300.68 ± 6.51 ab	118.73 ± 2.11 i	29.44 ± 2.17 cd	30.36 ± 0.85 f	0.12 ± 0.00 efg	27.42 ± 0.42 d	7.59 ± 0.14 f	51.43 ± 1.10 e	17.98 ± 0.69 de	0.19 ± 0.00 c	70.04 ± 0.16 f	6.94 ± 0.02 a
YJ2	32.05 ± 0.93 b	6.86 ± 0.19 cde	332.69 ± 10.40 ab	305.27 ± 11.13 ab	186.02 ± 3.43 a	29.84 ± 2.38 cd	26.75 ± 0.38 g	0.1 ± 0.02 fgh	37.98 ± 0.72 c	11.84 ± 0.34 b	66.35 ± 1.74 c	20.18 ± 0.87 c	0.28 ± 0.01 b	239.59 ± 0.85 a	1.27 ± 0.01 g
YJ3	29.85 ± 0.35 cd	7.23 ± 0.29 bc	317.17 ± 2.30 bc	244.12 ± 8.45 fg	142.24 ± 4.60 gh	34.76 ± 1.99 abcd	32.17 ± 0.76 def	0.14 ± 0.01 de	36.84 ± 1.38 c	8.3 ± 0.20 de	67.88 ± 0.51 bc	11.6 ± 0.12 g	0.19 ± 0.00 c	130.37 ± 0.59 c	0.15 ± 0.01 i
YJ4	25.39 ± 0.72 fg	6.10 ± 0.18 fgh	277.99 ± 5.81 ef	252.28 ± 3.45 ef	75.64 ± 1.59 k	36.52 ± 1.07 ab	40.36 ± 1.39 a	0.13 ± 0.01 ef	40.14 ± 1.79 c	3.66 ± 0.08 i	20.34 ± 0.39 h	12.88 ± 0.09 fg	0.28 ± 0.01 b	68.06 ± 0.33 fg	2.20 ± 0.03 e
YJ5	31.92 ± 0.17 b	7.30 ± 0.23 bc	263.83 ± 3.50 fg	235.98 ± 5.97 fg	164.65 ± 3.83 de	32.89 ± 2.68 abcd	29.69 ± 0.63 f	0.13 ± 0.02 ef	49.54 ± 1.92 b	8.37 ± 0.10 de	48.02 ± 0.59 e	19.92 ± 0.59 cd	0.19 ± 0.00 c	91.38 ± 0.33 e	2.17 ± 0.02 e
YJ6	39.28 ± 1.14 a	6.45 ± 0.29 def	245.68 ± 8.80 gh	200.29 ± 3.92 i	168.08 ± 3.47 cd	33.75 ± 2.92 abcd	24.78 ± 0.70 g	0.18 ± 0.01 ab	51.34 ± 1.77 b	6.22 ± 0.13 g	65.98 ± 1.66 c	14.32 ± 0.20 f	0.19 ± 0.00 c	157.93 ± 1.14 b	3.81 ± 0.09 b
YJ10	28.19 ± 0.45 de	6.34 ± 0.14 efg	264.15 ± 8.20 fg	312.74 ± 4.37 a	148.66 ± 1.66 g	33.68 ± 1.97 abcd	35.35 ± 1.03 bc	0.10 ± 0.00 gh	24.81 ± 1.19 de	8.56 ± 0.16 de	58.19 ± 0.49 d	20.42 ± 0.93 c	0.29 ± 0.01 a	129.61 ± 0.16 c	0.09 ± 0.04 i
YL1	28.66 ± 0.40 cde	5.75 ± 0.13 gh	287.14 ± 10.33 de	287.33 ± 1.78 bc	82.91 ± 1.84 jk	31.55 ± 2.03 bcd	36.98 ± 0.99 b	0.20 ± 0.01 a	24.61 ± 1.04 de	6.56 ± 0.05 g	26.82 ± 1.03 g	16.42 ± 0.62 e	0.19 ± 0.00 c	107.80 ± 0.16 d	1.60 ± 0.27 f
CK	25.32 ± 0.66 g	5.54 ± 0.17 hi	319.80 ± 10.73 bc	272.72 ± 6.60 cd	137.02 ± 3.21 h	32.96 ± 1.88 abcd	31.96 ± 0.95 ef	0.12 ± 0.01 efgh	36.36 ± 1.60 c	16.31 ± 0.39 a	51.47 ± 1.10 e	34.82 ± 1.30 a	0.19 ± 0.00 c	101.67 ± 1.02 de	3.67 ± 0.02 bc

SP, soluble protein; CFat, crude fat; TSt, total starch; TSu, total sugar; TSS, total soluble sugar; TKN, total Kjeldahl nitrogen; CF, crude fiber; CA, crude ash; Pec, pectin; TP, total polyphenols; TSs, total saponins; Flav, flavonoids; AA, ascorbic acid; CAT, catalase; POD, peroxidase. The same below.

**Figure 4:**
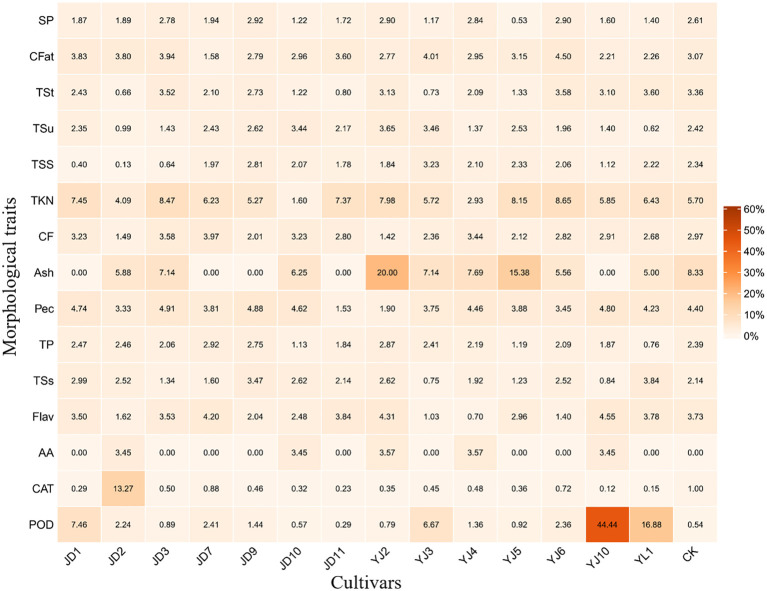
Coefficient of variation of pod and seed nutritional quality.

Among the nutritional quality traits across different cultivars (**Figure 4**), total Kjeldahl nitrogen (TKN) exhibited the highest average CV (6.13%), followed by POD (5.95%) and Ash (5.89%); ascorbic acid (AA) showed the lowest average CV at only 1.17%, followed by CAT (1.31%) and TSS (1.80%). Among all cultivars, YJ10 displayed the highest average CV (5.22%), followed by YJ2 (4.00%), while the remaining cultivars ranged from 2.01% to 3.59%. Compared to morphological traits, the CVs in nutritional quality were relatively lower, indicating a higher genetic stability.

### 3.3 Variable importance and correlation of different cultivars

**Figure 5:**
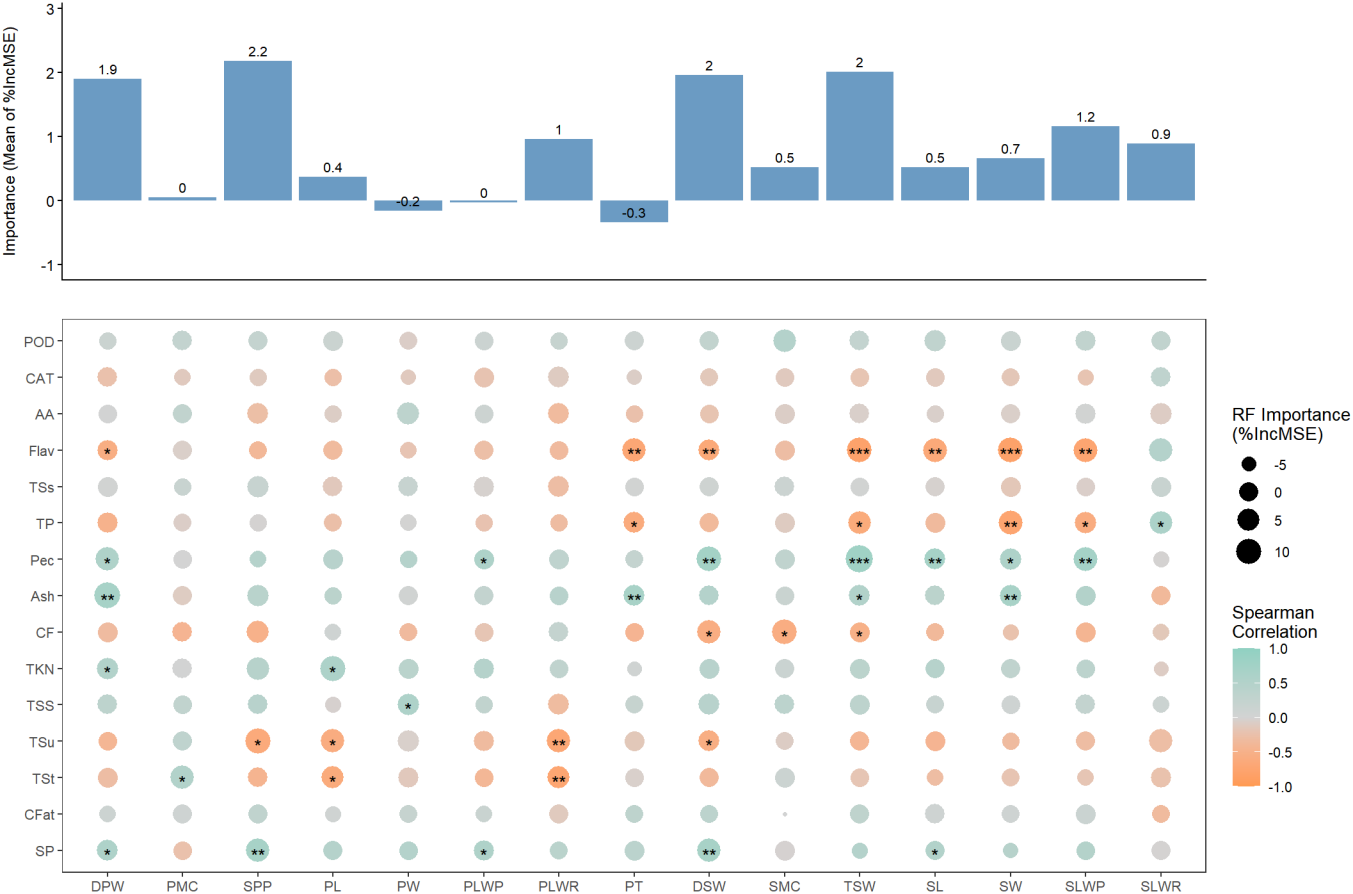
Random forest (top) and spearman correlation (bottom) of different trait indices. The circle colors indicate the direction of correlations (green: positive; red: negative). The symbols *, **, and *** denote significance levels at p< 0.05, p<0.01, and p< 0.001, respectively. The circle size indicates represents the magnitude of permutation importance of variables in the random forest model.

The Random Forest model assessed the contribution of each morphological trait to the prediction of nutritional quality indicators using the mean percent increase in mean squared error (%IncMSE) (**Figure 5**). The results revealed that SPP held the highest importance (2.2), followed by DSW (2.0), TSW (2.0), and DPW (1.9), indicating that these morphological traits contributed substantially to the model’s predictive accuracy. Conversely, PW (-0.2) and PT (-0.3) exhibited negative %IncMSE values, suggesting that these traits contributed marginally to the models and potentially introduced noise rather than improving prediction accuracy.

Spearman’s rank correlation analysis showed that Flav, TP, CF, and TSu were significantly negatively correlated with multiple morphological indicators (*P* < 0.05), whereas Pec, Ash, and SP were significantly positively correlated with multiple morphological indicators (*P* < 0.05). Specifically, Flav was significantly negatively correlated with DPW, PT, DSW, TSW, SL, SW, and SLWP; TP was significantly negatively correlated with PT, TSW, SW, SLWP, and SLWR; CF was significantly negatively correlated with DSW, SMC, and TSW; TSu was significantly negatively correlated with SPP, PL, PLWR, and DSW (*P* < 0.05). Pec was significantly positively correlated with DPW, PLWP, DSW, TSW, SL, SW, and SLWP; Ash was significantly positively correlated with DPW, PT, DSW, TSW, and SW; SP was significantly positively correlated with DPW, SPP, PLWP, TSW, and SL (*P* < 0.05). Additionally, TSt was significantly positively correlated with PMC, but significantly negatively correlated with PL and PLWR (*P* < 0.05).

### 3.4 PCA of pod and seed morphological traits across different cultivars

**Figure 6:**
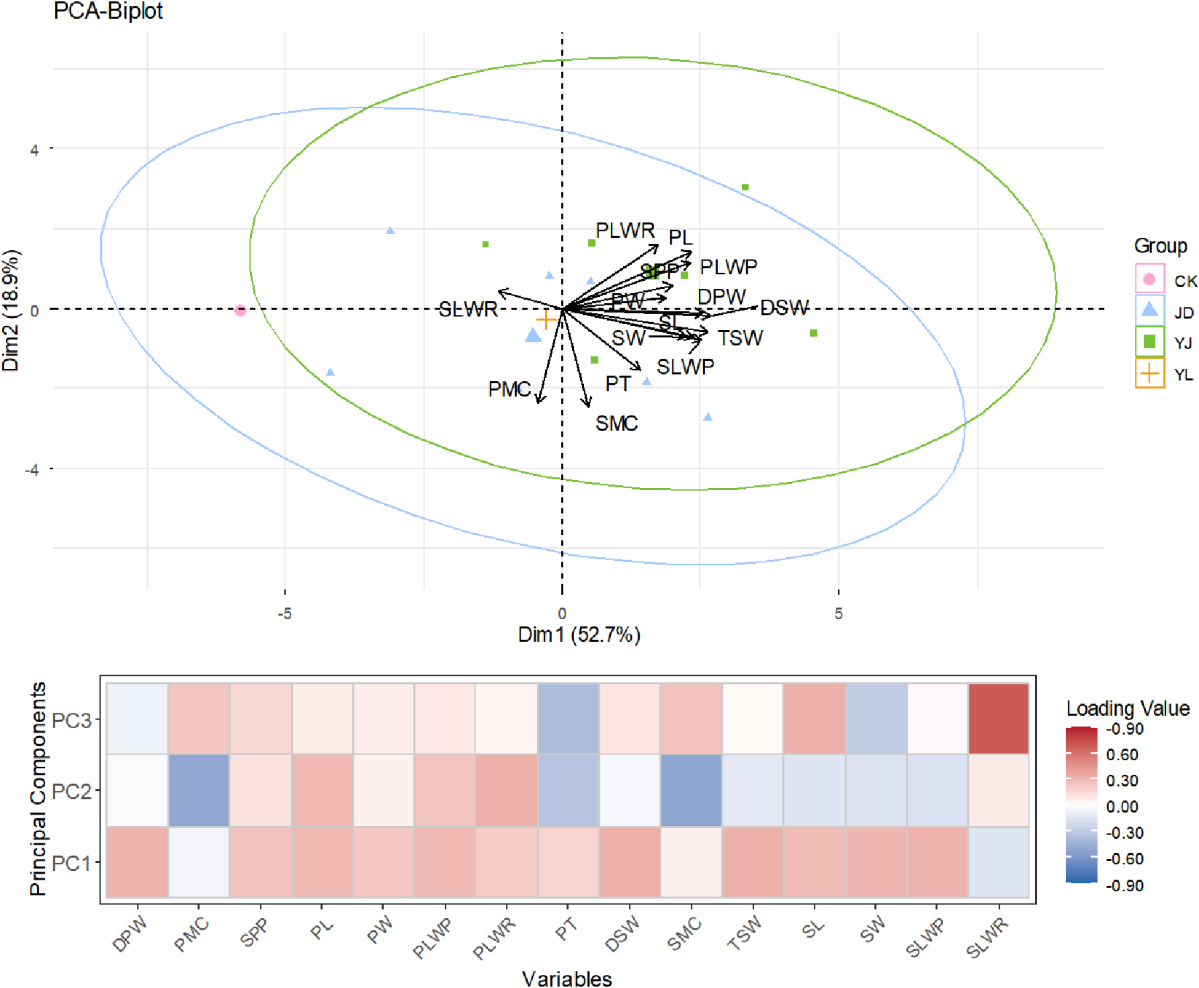
PCA and loadings of pod and seed morphological traits.

PCA of the 15 pod and seed morphological traits across different cultivars revealed that the first three principal components (PC1-PC3) cumulatively explained 81.72% of the total variance, adequately capturing the core information of the original traits (**Figure 6**). The PC1, accounting for 52.68% of the variance, was interpreted as a size-weight variation dimension. This interpretation is based on the traits with the highest positive loadings, such as DSW (0.338), DPW (0.322), SLWP (0.317), and TSW (0.333), which physically define the spatial dimensions and mass of the organs. Thus, PC1 logically reflects the overall developmental level of pods and seeds. The PC2, explaining 18.94% of the variance, represented a moisture characteristic dimension. It was primarily loaded by PMC (-0.500) and SMC (-0.516), which directly measure water content, along with PLWR (0.336) and PT (-0.327). The independence of PC2 from PC1 indicates that moisture content was not constrained by size and weight.

The remaining components captured more specific morphological variations. PC3 (10.10% of the variance) represented a seed shape dimension, primarily loaded by SLWR (0.686) with secondary contributions from SL (0.336) and PT (-0.375). Since SLWR and SL inherently define the geometric proportions of the seeds, PC3 reflects specific morphological differentiation in seeds. Together, these dimensions elucidate the multi-dimensional phenotypic divergence among the cultivars.

### 3.5 PCA of pod and seed nutritional quality traits across different cultivars

**Figure 7:**
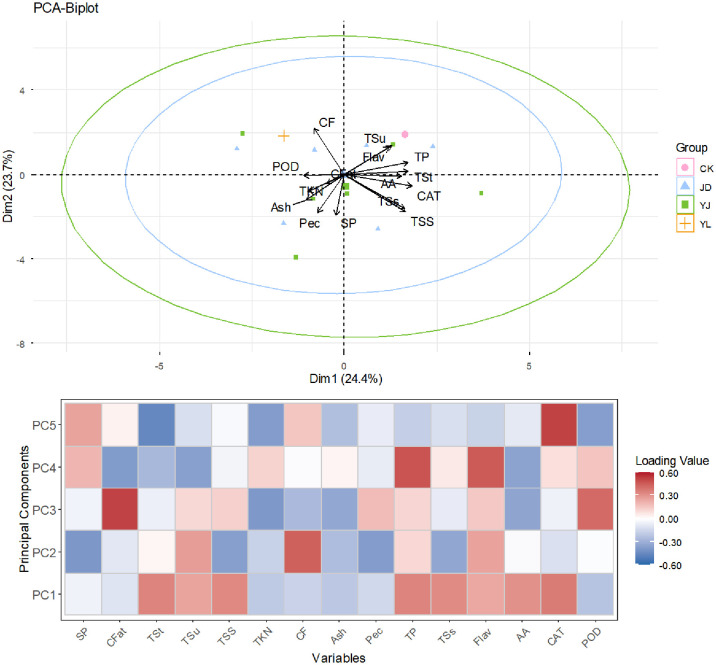
PCA and loadings of pod and seed nutritional quality.

PCA of the 15 pod and seed nutritional quality traits across different cultivars revealed that the first five PCs (PC1–PC5) cumulatively explained 81.27% of the total variance (**Figure 7**). Notably, the first two PCs, with a cumulative contribution of 48.05%, effectively captured the core variation information and broadly reflected the differences in nutritional quality among cultivars. PC1, accounting for 24.37% of the variance, constituted a core dimension for basic and textured nutrition. It was predominantly loaded by CAT (0.367), TP (0.346), TSt (0.345), TSS (0.331), TSs (0.326), and AA (0.310). As TSS, TSs, and TSt represent primary soluble nutrients and starch, their joint loadings reflect the coordinated variation of soluble nutrients and certain functional active compounds. PC2 (23.69% of the variance) formed a dimension of functional activity and flavor nutrition. Its core loadings included CF (0.441), SP (-0.395), TSS (-0.362), Pec (0.374), TSs (0.335),. Given that Flav and TP are well-known functional active compounds, while TSu and CF relate to flavor and taste, this combination indicates a synchronous accumulation trend between functional active compounds and flavor-related substances.

The remaining PCs captured more specific nutritional variations. PC3 (13.82% of the variance) served as a supplementary dimension for fat and mineral nutrition, primarily loaded by CFat (0.520), POD (0.418), TKN (-0.389), AA (-0.340), Ash (-0.331) and. Since CFat directly measures lipid content, while Ash and TKN are standard indicators of mineral content, this component independently elucidates the divergent variation patterns of lipid components and mineral nutrients. PC4 (11.52% of the variance) further refined the variation associations between functional active compounds and lipid components. It was mainly loaded by TP (0.476), Flav (0.456), CFat (-0.380), TSu (-0.359), and AA (-0.345). Because TP and Flav are functional metabolites that inversely co-vary with CFat (lipids) and AA, this reflects the synergistic accumulation of functional active compounds alongside an evident negative trade-off with crude fat content. Finally, PC5 (7.88% of the variance) constituted a dimension for enzyme activity and starch variation. Driven primarily by CAT (0.521), TSt (-0.457), TKN (-0.372), and POD (-0.365), as CAT and POD are key antioxidant enzymes, and total starch (TSt) measures starch, their grouping reflects the independent variation between enzymatic activities and starch content.

### 3.6 Comprehensive evaluation of pod and seed traits across different cultivars

Comprehensive evaluation using the membership function method based on the first five PCs (**Table 4**) revealed that across the 14 tested cultivars, the M values ranged from 0.15 to 0.71, the N values ranged from 0.24 to 0.61, and the C values ranged from 0.34 to 0.55, indicating significant differences in pod and seed traits. Within the JD series, JD1 and JD7 exhibited advantages only in nutritional quality, whereas JD2 and JD3 showed superiority solely in morphological traits. JD9 and JD10 performed moderately in both aspects, while JD11 performed poorly in both. In the YJ series, YJ2 demonstrated a distinct advantage only in nutritional quality; YJ4, YJ5, and YJ6 excelled exclusively in morphological traits; YJ3 exhibited balanced but not outstanding performance across both aspects (M = 0.50, Q = 0.36, C = 0.43); and YJ10 showed moderate overall performance with relatively better nutritional quality (M = 0.43, Q = 0.51, C = 0.47). Additionally, YL1 showed a balanced development in both morphology and nutritional quality. In summary, YJ5, YJ2, JD7 and YJ6 were screened as elite cultivars with superior comprehensive traits. For CK, the C value was 0.40, with a relatively low M (0.19) and a moderate N (0.61), indicating that it possessed relatively good nutritional quality but poor morphological development of pods and seeds.

**Table 4:** Comprehensive evaluation of different cultivation varieties.

Sp	M	N	C	Rank
JD1	0.15	0.53	0.34	15
JD2	0.49	0.27	0.38	12
JD3	0.48	0.24	0.36	14
JD7	0.38	0.59	0.49	3
JD9	0.52	0.43	0.48	5
JD10	0.46	0.39	0.42	9
JD11	0.34	0.46	0.40	10
YJ2	0.45	0.56	0.50	2
YJ3	0.50	0.36	0.43	8
YJ4	0.59	0.32	0.45	7
YJ5	0.71	0.39	0.55	1
YJ6	0.69	0.28	0.49	4
YJ10	0.43	0.51	0.47	6
YL1	0.36	0.40	0.38	13

M, N and C respectively represent the degree of morphology membership, nutritional membership, and comprehensive membership for pod and seed.

### 3.7 Cluster analysis of pod and seed traits across different cultivars

**Figure 8:**
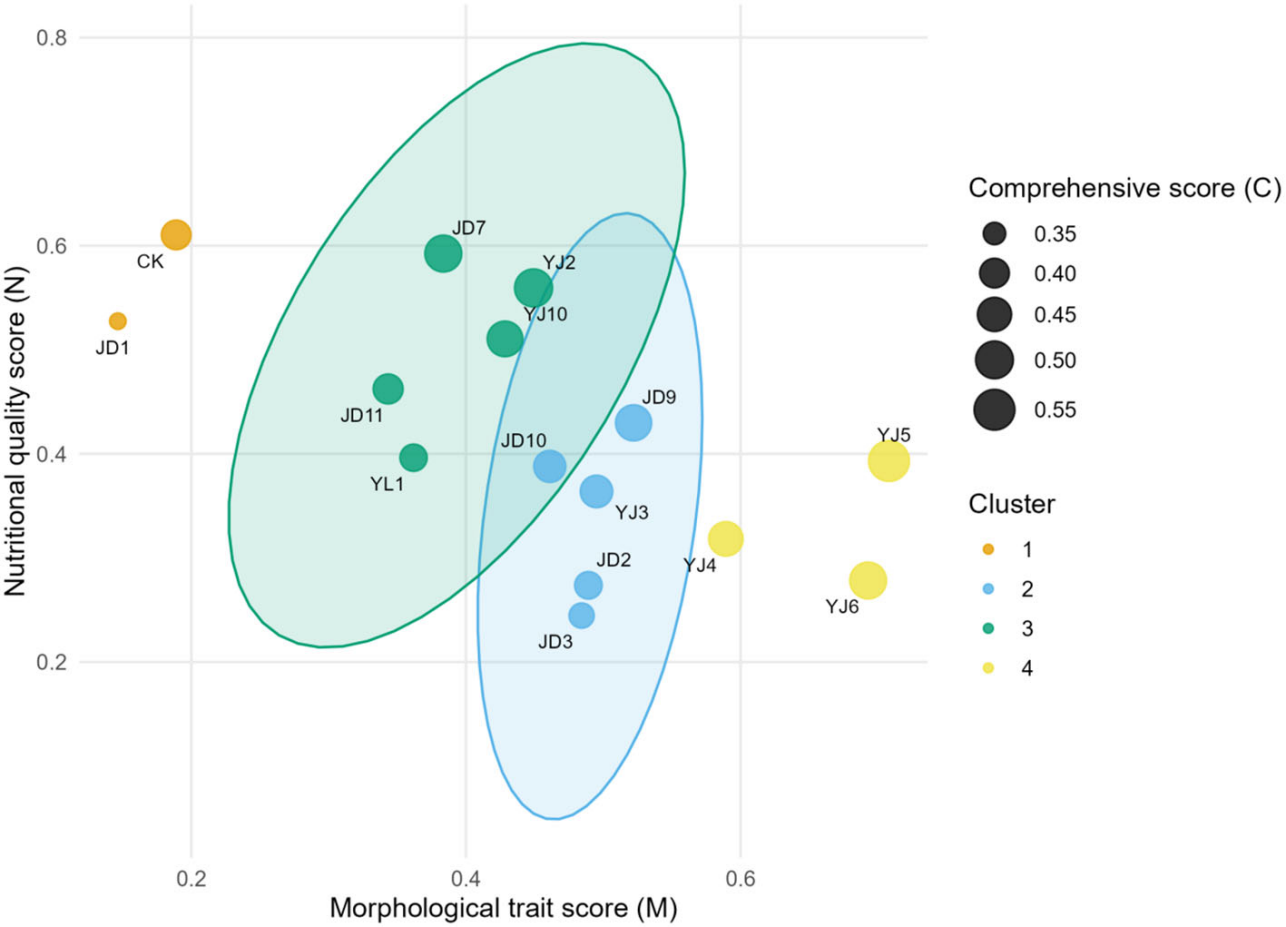
Hierarchical clustering analysis of different cultivar samples based on M and N.

Based on the hierarchical cluster analysis of morphological (M) and nutritional quality (N) traits, the 14 cultivars were divided into four distinct clusters (**Figure 8**).

Cluster I contained three cultivars, including YJ5, YJ4, and YJ6, representing a morphological advantage-nutritional disadvantage type. This group had relatively high M values (0.59–0.71) but moderate N values (0.28–0.39), with a mean C of 0.49. Notably, YJ5 (C = 0.55) and YJ6 (C = 0.49) displayed superior morphological traits, making them ideal materials for improving morphological characteristics.

Cluster II comprised five cultivars, including JD2, JD3, JD9, JD10, and YJ3, representing a morphological advantage-medium nutritional type. This cluster had moderate N values (0.24–0.46) and M values (0.34–0.50), with a mean C of 0.42. Notably, YJ3 (C = 0.43), JD9 (C = 0.48) and JD10 (C = 0.42) showed balanced performance in both dimensions, making them valuable materials for breeding.

Cluster III consisted of the wild species CK and JD1, defined as a morphological disadvantage-nutritional advantage type. It was characterized by extremely low M values (mean 0.17) but relatively high N values (0.53-0.61). It is particularly noteworthy that CK, as a wild species, was constrained by wild traits, resulting in small pods or seeds and poor morphological performance (M = 0.14). However, natural evolution endowed it with a robust capacity for nutrient accumulation (N = 0.61). Harboring elite genes absent in cultivated varieties, CK serves as a precious donor parent for improving nutritional quality.

Cluster IV included five cultivars, including JD7, YJ10, YJ2, JD11 and YL1, representing a nutritional advantage-medium morphological type. This group had moderate M values (0.35–0.45) and relatively high N values (0.46–0.59), with a mean C of 0.47. Notably, JD7 (C = 0.49), YJ10 (C = 0.47), YJ2 (C = 0.50), JD11 (C = 0.40), and YL1 (C = 0.38) displayed balanced performance in both dimensions, making them valuable breeding materials.

The results of the hierarchical cluster analysis and the membership function method were both consistent and complementary. Their consistency lies in the general alignment between the C value ranking and the cluster partitioning, with both methods identifying germplasm with comprehensive advantages. Specifically, Cluster I and IV both contained cultivars with high C values (e.g., YJ5 in Cluster I with C = 0.55 and YJ2 in Cluster IV with C = 0.50), while Cluster II and III had relatively lower C values. The difference, however, stemmed from the fact that the C value is a weighted mean, which can easily mask single-dimensional characteristics. For example, the high M value of YJ5 inflated its C value ranking, thereby obscuring its nutritional deficiency (N = 0.39). More critically, the wild species CK yielded a low C value (0.40) due to its extremely low M value (0.19); relying solely on the C value would likely lead to its elimination. In contrast, the cluster analysis, combined with its wild background, accurately highlighted the unique value of CK’s high-nutrition genes (N = 0.61). Combining these two methods authentically reveals the phenotype-nutrition trade-offs, avoids the blindness of single-indicator evaluation, effectively mines specific germplasm like CK, and provides a reliable basis for precision breeding.

## 4 Discussion

Common garden experiments are invaluable for assessing adaptive genetic variation free from the confounding effects of environmental variation and phenotypic plasticity (Schwinning et al., 2022; Huxman et al., 2021; De Villemereuil et al., 2020). By growing 14 main cultivars of *G. sinensis* (including Jindou, Yujia, and Yulin series) and a wild control under identical conditions, this study effectively stripped away the interference of environmental variance (G × E interaction), attributing phenotypic differences directly to genotypic variations (De Villemereuil et al., 2020). Importantly, these cultivar series were primarily developed through phenotypic selection of superior individuals from distinct geographical populations without complex pedigree crosses, which provides the necessary biological context for the underlying genetic diversity and phenotypic variation observed. Phenotypic traits, arising from gene-environment interactions, are fundamental for germplasm evaluation and partially reveal genetic diversity (Luo et al., 2025; Hu, 2009). The coefficient of variation (CV) directly reflects this variation, with higher values indicating greater variability and richer phenotypic diversity (Li et al., 2020b; Luo et al., 2025). The CV analysis revealed a highly instructive pattern: the average CVs for seed size and morphological traits were smaller than those for pods, indicating greater variability in pod characteristics and higher stability in seed traits (Luo et al., 2025). Furthermore, the CV amplitude of morphological traits (e.g., SPP 29.62%, DPW 23.13%, PMC 22.96%) was much higher than that of nutritional quality indicators (e.g., AA 1.17%, CAT 1.31%, TSS 1.80%). This indicates that while the morphology of *G. sinensis* retained high plasticity to adapt to changing environments, biochemical indicators are subject to stricter genetic regulatory network constraints, exhibiting high stability. This has important breeding implications: although morphological traits are susceptible to environmental influences (Luo et al., 2025; Xiao et al., 2024), their genetic potential can be efficiently excavated; and the strong genetic stability of functional components ensures the reproducibility of excellent quality in different environments.

Comparing the wild type (CK) with cultivated varieties reveals a clear artificial domestication trend. Cultivated varieties (i.e., Yujia and Yulin series) significantly outperformed CK in yield traits (e.g., pod dry weight, 1000-seed weight) and total saponin content, successfully achieving the breeding goal of “large pods, heavy seeds, and high saponins” (Singh and Van Der Knaap, 2022; Figueroa-Macías et al., 2021). However, CK accumulated significantly higher total polyphenols (TP) and flavonoids (Flav), aligning with findings that artificial domestication, while increasing yield, is often accompanied by a decline in intrinsic plant defense substances (Strange and Scott, 2005; Bautista et al., 2015; Mansoor et al., 2023). In artificial cultivation environments, pesticide protection replaces chemical defense needs, leading to the suppression or relaxation of defense-related gene expression during domestication, known as the domestication syndrome (Singh and Van Der Knaap, 2022). Notably, saponin content did not decrease in cultivated varieties, potentially due to its dual function in defense and growth as adaptogenic substances (Lian and Zhang, 2013; Deng et al., 2024). A key finding is the significant negative correlation between biomass accumulation indicators (e.g., DPW, TSW) and secondary metabolites (e.g. TP, Flav). While these statistical correlations do not directly establish physiological trade-offs or causal resource allocation mechanisms, they suggest a potential growth-defense trade-off that aligns with theoretical carbon allocation expectations. Theoretically, cultivated varieties allocate more assimilates to primary metabolism, the expanded pods and seeds constitute extremely strong metabolic sinks, intercepting photosynthates for structural carbohydrates, competitively reducing substrates available for secondary metabolism (De Casas et al., 2024; Parveen et al., 2025; Wu et al., 2025a, 2025). For example, JD3 exhibited high pod dry weight (42.41 ± 10.2 g), but low total polyphenol content (4.85 ± 0.10 mg/g) and CAT enzyme activity, whereas CK appears to have directed more resources toward secondary metabolism. To fully elucidate this resource allocation mechanism, future physiological, biochemical, and molecular studies are required.

These strong phenotype-metabolite associations also offer practical predictive value for breeding. Breaking the traditional reliance on high-cost technologies (e.g., NIRS, multispectral drone imaging, deep learning) to independently predict morphological or nutritional traits (Martínez-Valdivieso et al., 2018; Zhang et al., 2025b). The random forest analysis revealed that low-cost, easily measurable morphological indicators (i.e., SPP, DPW, and TSW) hold extremely high predictive importance for internal nutritional quality. Seed yield is determined by quantitative traits like pod number per plant (PN), SPP, and seed weight (SW) (Xin et al., 2021; Luo et al., 2025). which possess relatively high heritability (Lu et al., 2017; Shi et al., 2015) and weak trade-offs with each other (Shi et al., 2015; Zhu et al., 2020). SPP holds the highest predictive importance because more seeds intensify competition for primary nutrients, inevitably squeezing the allocation to secondary metabolites. Furthermore, the pod length-to-width ratio (PLWR) is highly significantly negatively correlated with total polyphenols. Slender pods often retain more wild traits with higher polyphenol retention, whereas plump pods, products of intensity domestication, have increased biomass but decreased polyphenols. Therefore, these morphological markers enable rapid, low-cost preliminary screening of early hybrid progeny for specific purposes. If breeders aim to extract polyphenols and flavonoids, they can moderately relax selection thresholds for pod width; conversely, for industrial raw materials, plump pod germplasm should be selected (Ellis et al., 2021).

Based on a multidimensional evaluation system driven by the need to select genetic variability for unique or superior traits in agricultural applications (Fazwa Md Ariff et al., 2021), the germplasms were clearly differentiated into four clusters to guide differentiated utilization strategies: Cluster I (YJ5, YJ4, YJ6), Cluster II (JD2, JD3, JD9, JD10, YJ3), Cluster III (CK, JD1), and Cluster IV (JD7, YJ10, YJ2, JD11, YL1). JD9, YJ2, and YJ10 demonstrated balanced agronomic and nutritional performance, making them promising comprehensive cultivars. Although CK’s extremely low morphological score (0.19) might risk its elimination in single-dimensional evaluations, clustering precisely highlights its high nutritional potential (N = 0.61), confirming its irreplaceability as a crucial donor parent for future nutritional improvement. Conversely, YJ5 and YJ6 are ideal materials for enhancing morphological characteristics. The yield focus of modern breeding has narrowed the genetic base (Wang et al., 2020); utilizing the superior high-polyphenol/flavonoid alleles of CK is crucial for breaking the yield-quality trade-off via marker-assisted selection.

Although the common garden design effectively excluded environmental noise (Elias et al., 2016; Pramanik et al., 2024), this study has limitations. The data originate only from a single common garden and harvest year, without considering tree age effects or extreme fluctuations that might dynamically alter carbon allocation and trade-off intensity. Furthermore, the interpretation of the underlying trade-off is currently limited to phenotypic and biochemical associations. Future research requires multi-location, multi-year joint testing to verify the stability of core traits and trade-off relationships (G × E interaction effects). More crucially, in-depth analysis should be combined with multi-omics approaches to fundamentally achieve synergistic improvement of *G. sinensis* yield and quality (Wang et al., 2020), such as utilizing transcriptomics to mine key regulatory genes (e.g., MYB or bHLH families) (Xiao et al., 2025; Zhu et al., 2014), metabolomics to track the carbon flow reconfiguration (Wu et al., 2025b), and genome-wide association studies (GWAS) to locate QTLs controlling polyphenol and saponin synthesis (Lu et al., 2017; Xin et al., 2021).

## 5 Conclusion

This study systematically compares the morphological and nutritional quality differences of pods and seeds between 14 *G. sinensis* cultivars and the wild type under a common garden condition for the first time, confirming that these trait variations are primarily genetically determined. Notably, phenotypic variation was greater in pods than in seeds, and morphological variation of both pods and seeds exceeded that of nutritional quality, indicating that the latter exhibits higher genetic stability. Cultivars exhibit significant domestication advantages in pod dry weight, 1000-seed weight, and total saponin content, whereas the wild type retains a naturally evolved accumulation advantage in secondary metabolites such as total polyphenols and flavonoids. A negative correlation was observed between reproductive growth (larger/heavier pod and seed structures) and the accumulation of secondary metabolites (polyphenols and flavonoids), which potentially suggests a growth-defense trade-off during pod and seed development; furthermore, key morphological indicators (e.g., seeds per pod, seed dry weight, and 1000-seed weight) can serve as reliable predictors of internal quality. Based on a multidimensional evaluation system, the germplasms are clearly differentiated into four clusters: Cluster I (YJ5, YJ4, YJ6), Cluster II (JD2, JD3, JD9, JD10, YJ3), Cluster III (CK, JD1) and Cluster IV (JD7, YJ10, YJ2, JD11, YL1). Notably, JD9, YJ2, and YJ10 show balanced performance; the wild type (CK) and JD1 serve as crucial donor parents for future nutritional improvement, while YJ5 and YJ6 are ideal materials for improving morphological characteristics. Ultimately, these findings provide insights into the potential growth-defense trade-off during woody plant domestication, providing a scientific basis for the differentiated directional breeding and wild gene utilization in *G. sinensis*.

## Data Availability

The original contributions presented in the study are included in the article/supplementary material. Further inquiries can be directed to the corresponding author.
